# Smokers with higher positive or negative urgency have lower rates of smoking cessation success 12 months after a quit attempt

**DOI:** 10.1038/s41598-024-62972-6

**Published:** 2024-05-29

**Authors:** Paul Brunault, Isabelle Ingrand, Marcello Solinas, Emilie Dugast, Marie-Christine Pérault-Pochat, Pierre Ingrand, Paul Vanderkam, Claire Lafay-Chebassier

**Affiliations:** 1 CHRU de Tours, Service d’Addictologie Universitaire, Équipe de Liaison et de Soins en Addictologie, Tours, France; 2 UMR 1253, iBrain, Université de Tours, Inserm, Tours, France; 3 Université de Tours, QualiPsy, EE 1901, Tours, France; 4 Registre Des Cancers Poitou-Charentes, Université de Poitiers, Poitiers, France; 5 INSERM U-1084, Laboratoire de Neurosciences Expérimentales et Cliniques, Université de Poitiers, Poitiers, France; 6 Unité de Recherche Clinique Pierre Deniker, Centre Hospitalier Henri Laborit, Poitiers, France; 7 INSERM, Centre d’Investigation Clinique CIC 1402, Université de Poitiers, CHU Poitiers, Poitiers, France; 8 Service de Pharmacologie Clinique, CHU Poitiers, Poitiers, France; 9 Département de Médecine Générale, Université de Bordeaux, Bordeaux, France; 10 Centre Hospitalier Universitaire de Poitiers, 2 rue de la Milétrie – CS 90577, 86021 Poitiers Cedex, France

**Keywords:** Neuroscience, Medical research

## Abstract

Impulsivity dimensions have been shown to be associated with smoking status and tobacco use disorder severity. However, it is important to determine the specific impulsivity traits associated with smoking relapse. This study aimed at investigating the associations between impulsivity traits and smoking cessation success among adult smokers at 12 months after a quit attempt. Participants were 68 adult smokers enrolled in a 3-month course of simvastatine or placebo associated with behavioral cessation support, with a 9-month follow-up (ADDICSTATINE study). They were classified in 3 groups according to smoking status: abstinent, reduction ≥ 50%baseline or reduction < 50%baseline at 3 and 12 months. Impulsivity traits were assessed using the UPPS-P-scale. At 12 months, abstainers and participants who reduced smoking by 50% or more had significantly lower scores in negative and positive urgency compared to participants who reduced smoking by less than 50% (*p* = 0.011 and 0.0059). These urgency traits scores at 12 months were significantly and negatively correlated with smoking reduction at 12 months (*p* = 0.017 and 0.0012). These impulsivity traits were also associated with the smoking cessation success at 3 months. Patients who were abstinent at 3 months had also lower negative and positive urgency (*p* = 0.017 and 0.0039). Smoking cessation success at 3 and 12 months were not associated with the other impulsivity traits, sensation seeking, lack of premeditation or perseverance. Our findings suggest that positive and negative urgency are associated with smoking cessation success. Proposing better tailored-based-treatment targeting these impulsivity traits in combination with conventional treatment may help improving smoking treatment success.

## Introduction

According to the World Health Organization, smoking is the largest preventable cause of disease and death in the world^1^. The benefits of smoking cessation have been clearly proven in terms of morbidity and mortality for different diseases related to tobacco, especially for lung cancer^2^, but smokers quit and relapse several times before they eventually achieve sustained abstinence^3^. Relapse of smoking after attempting to quit most frequently occurs within the first few weeks^4^. Within the first 12 months of smoking abstinence, risk of relapse is more than 50%^5^. Considering these high relapse rates, it is critical to understand the risk factors that predict the success or failure of cessation attempts.

Several risk factors for smoking relapse have been identified: higher severity of nicotine dependence, higher severity of withdrawal or craving symptoms, as well as higher number of years smoking, previous quit attempts and length of abstinence^6^ and a history of any psychiatric disorder, especially the co-occurrence with other substance use disorders^7^. Individual differences in some personality traits also appear to increase susceptibility to relapse^8^. Among these factors, impulsivity is considered as a major risk factor for smoking behavior, smoking relapse^8^ and cigarette craving^9^.

Impulsivity can be defined as the tendency to engage rapidly in a behavior without appropriately considering the potential negatives consequences of the behavior^10^. Among the different models used to assess impulsivity-related constructs, we chose to focus here on the UPPS-P model developed by Whiteside and Lynam^11^ and revised by Cyders and Smith^12^ because it considers impulsivity as a multidimensional construct, because it has demonstrated its clinical relevance in persons with addictive disorders^13,14^ and because it assesses specifically emotion-related impulsivity, a core risk factors for addictive disorders and externalizing behaviors^15,16^. According to these authors Whiteside and Lynam^11^ and Cyders and Smith^12^, impulsivity can be described as a multidimensional personality trait that comprises five different interrelated traits: positive urgency (i.e., tendency to act rashly in response to intensely positive emotional states), negative urgency (i.e., tendency to act rashly when distressed), sensation seeking (i.e., tendency to seek sensory pleasure, excitement and novel experiences), lack of premeditation (i.e., tendency to act without forethought) and lack of perseverance (i.e., inability to remain focused on a task and to quit when a task becomes difficult or boring). These five impulsivity facets can be assessed using the validated UPPS-P Impulsive Behavior Scale that is extensively used in patients with addictive disorders, including tobacco^9,13,16^. According to some authors, the UPPS-P has the potential to characterize the influence of these distinct aspects of impulsivity on addictive behaviors^17^.

However, we lack an understanding of the extent to which each of the five dimensions of impulsivity may be differentially associated with smoking withdrawal and cessation outcome. Data were limited for a number of traits analysed. Martin-Rios et al. showed that self-reported impulsivity is directly related to the risk of relapse after a prolonged period of clinical follow-up of 12 months and conclude that it is essential to determine the role of each impulsive component during the abstinence process^18^. In a recent meta-analysis^19^, the majority of the included studies have assessed sensation seeking and lack of premeditation, and few studied the urgency traits, that are more directly linked to emotion regulation, and specifically positive urgency. They showed that all traits in the UPPS-P model were positively associated with smoking status, with small weighted mean effect sizes. Lack of premeditation and positive urgency showed the largest mean associations but the confidence intervals overlapped with those for all other UPPS-P traits. One of the limitations put forward by Kale et al. is that only 3 studies assessed positive urgency and most of the studies involve non-clinical populations and not involved in the smoking cessation process. Finally, impulsivity assessments were obtained at treatment onset, and not after post-treatment or after acute and chronic tobacco abstinence. In sum, there is a need for more research to determine what are the specific impulsivity traits associated with smoking relapse in clinical population including positive and negative urgency. This may help us improving smoking treatment success by proposing better tailored-based treatment targeting these corresponding impulsivity traits. In this study, we chose to focus more specifically on emotion-related impulsivity (i.e., negative urgency and positive urgency) because it explains variance in externalizing behaviors beyond that accounted for by other personality factors that corelate strongly with neuroticism^15^.

Therefore, the main objective of this study was to investigate the associations between impulsivity traits and smoking cessation success among adults’ smokers at 12 months after a quit attempt. Our secondary objective was to determine what other tobacco associated factors assessed at three months after the beginning of the cessation attempt, were associated with impulsivity traits.

## Methods

### Study design

The ADDICSTATINE study was a randomized, parallel-group, double blinded, placebo-controlled clinical trial assessing the efficacy of simvastatin in smoking cessation (ClinicalTrials.gov NCT02399709 registered on 26/03/2015). All methods were performed in accordance with the relevant guidelines and regulations. It was approved by the French Regional Ethics Committee (06/01/2015 n°2014-004978-42) and Drug Regulatory Agency (25/02/2015 n°141558A-32), and conducted at the Clinical Investigation Center of the University Hospital of Poitiers (France) between April 2015 and November 2016. The sample consisted of 118 participants (smokers) who received behavioral cessation support every 2 weeks by trained research nurses and were randomly assigned to a 3-month treatment period with either simvastatin or placebo, with a 9-month follow-up. Informed consent was obtained from all subjects. We calculated that based on a 25% expected detectable difference in efficacy on smoking reduction or abstinence between simvastatin and placebo, 80% power was achieved with 50 evaluable patients in each group (20% placebo vs 45% simvastatin, one-sided 5% exact Fisher test). The primary outcome was self-reported abstinence or smoking reduction ≥ 50% during the week before the 3-month follow-up visit and the secondary outcomes measures included self-reported prolonged abstinence at months 6 and 12-post-Target Quit Date. Results were described in detail previously^20^. Briefly, participants in the simvastatin group (n = 58) and the placebo group (n = 60) did not differ significantly at baseline according to demographic and smoking-related variables. Simvastatin treatment was not associated with higher 3-month abstinence or smoking reduction compared to placebo. At 12 months, smoking remained significantly reduced from baseline but was not significantly different in both groups (simvastatin 14%, placebo 15%).

One of the secondary aims of the ADDICSTATINE study was to investigate the possible psycho-socio-environmental risk factors including impulsivity traits that could be associated to inter-individual variability in smoking cessation and relapse. The present work reports the data obtained from the 68 participants who finished the study and completed the impulsivity scale (patients of simvastatin and placebo groups were pooled in this work).

### Participants and procedure

Eligible men and women aged 18–70 years were randomly assigned to receive either 40 mg orally once a day simvastatin or matched placebo for 3 months in addition to individual and personalized behavioral cessation support. Inclusion criteria were daily smoking ≥ 10 cigarettes/day for at least 1 year and wishing to quit smoking. Exclusion criteria were contraindication to simvastatin, active treatment by lipid-lowering agent, existence of one of the following psychiatric disorder (i.e., current major depressive disorder, current psychotic disorder, current cognitive disorders, mental retardation), a co-occurrent substance use disorder such as alcohol use disorder or abuse of substances other than tobacco, ≥ 3-months cigarette smoking abstinence in the previous year, use of smoking-cessation medication (Nicotine Replacement Therapy, bupropion, varenicline) or undergoing cognitive-behavioral therapy or use of clonidine or nortriptyline or electronic cigarette for smoking cessation in the last 3 months, pregnancy, breast-feeding and women of childbearing potential without adequate method of contraception. The existence of psychiatric and addictive disorders was assessed using a clinical interview based on DSM-IV-TR criteria conducted by the investigating physician.

**Figure 1:**
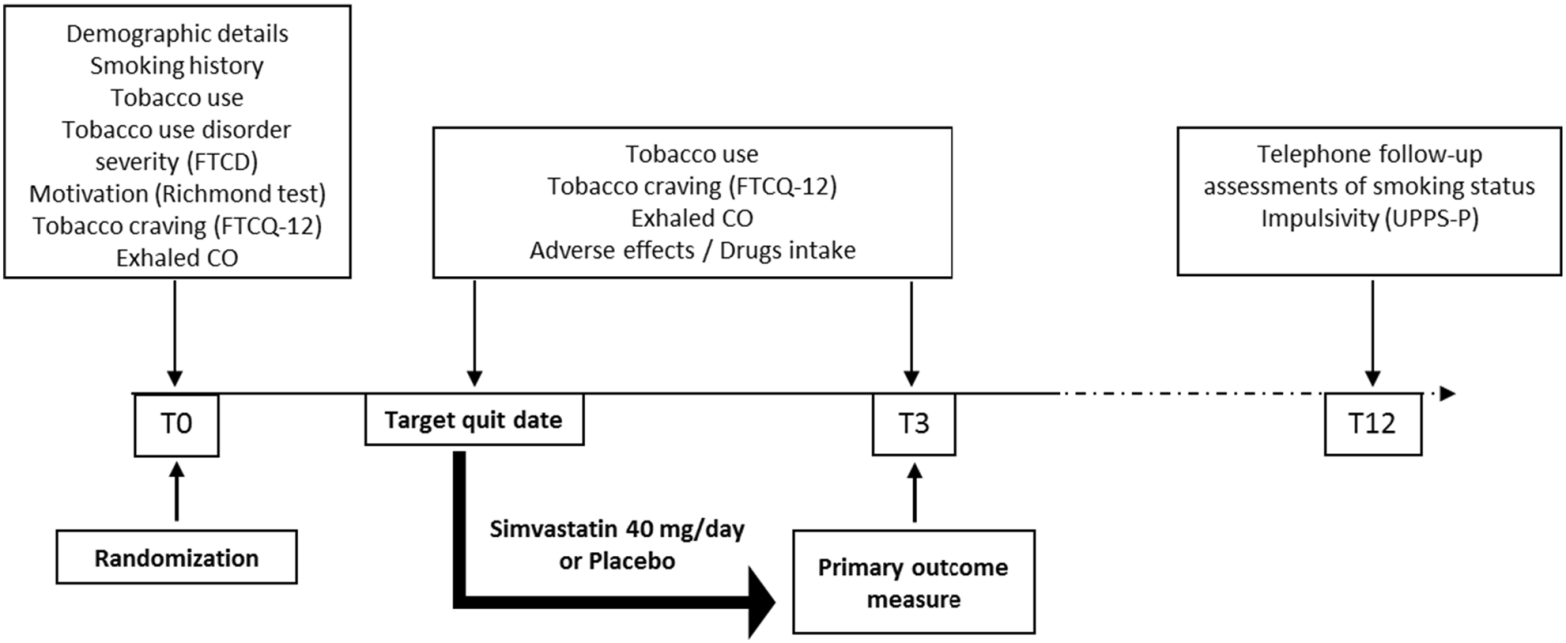
Study procedures of the ADDICSTATINE study.

Study procedures of the ADDICSTATINE study are presented in Fig. 1.

At baseline (just before the beginning of the cessation attempt, T0), data on demographic, medical history, smoking history, cigarette dependence severity and motivation to quit were collected. At month 3 (T3) study visit, we recorded data on craving, exhaled carbon monoxide concentration (MicroCO^®^, Eolys, Lyon, France), body weight and all adverse events. Participants were contacted at month 12 (T12) post-treatment of smoking cessation for blinded telephone follow-up assessments of smoking status (abstinent, reduction > = 50% baseline or reduction < 50% baseline). Each participant completed the UPPS-P at T12.

### Measures

#### Smoking status and tobacco dependence

At T0, we assessed tobacco dependence using the Fagerström Test for Nicotine Dependence (FTND), also referred as the Fagerström Test for Cigarette Dependence FTCD (original version: ^21^; French validation: ^22^). The FTND is composed of 6 questions assessing nicotine/tobacco dependence and its severity.

At T3 and T12, participants were classified in one of the two following groups: abstinent, non-abstinent (reduction in the number of cigarettes smoked > = 50% baseline or reduction in the number of cigarettes smoked < 50% baseline). At T3, participants were considered abstinent if they self-reported abstinence for 7 days before the assessment and provided a breath sample with a carbon monoxide concentration of less than 8 ppm. At T12, participants were considered abstinent if self-reported prolonged abstinence.

#### Motivation to quit smoking

The assessment of motivation to quit smoking was performed using the Richmond test^23^. This is a 4-item questionnaire with a total score ranging between 0 and 10. Scores between 0 and 5 indicate none or low motivation; 6–7 moderate motivation with a need for help and over 8 high motivation to quit smoking.

#### Craving

Craving for tobacco was assessed by the 12-item French Tobacco Craving Questionnaire (FTCQ-12,^24^). Items of FTCQ-12 were rated on a 7-item Likert scale (strongly disagree to strongly agree). The FTCQ-12 general craving score was obtained by the sum of each item’s score divided by the total number of items. The evolution of the FTCQ-12 between baseline and 3-months was the difference between the value at T3 and T0.

#### Impulsivity

Impulsivity facets were assessed at T12 using the French validated version of the UPPS Impulsive Behavior Scale, short version (UPPS-P)^25^. The UPPS-P is a 20-item self-administered questionnaire that is based on the UPPS ^11,26^ and one measure of positive urgency^12^. As described in the Introduction, the UPPS-P assesses five impulsivity facets: negative urgency (four items); positive urgency (four items), lack of premeditation (four items), lack of perseverance (four items), and sensation seeking (four items)^27^. Each item is assessed using a four-point Likert scale ranging from one (“agree strongly”) to five (“disagree strongly”). The UPPS-P enables the calculation of sub-score for each impulsivity facet, with higher scores indicating a higher intensity of the facet under consideration (items belonging to the positive urgency, negative urgency, and sensation seeking subscales are reversely scored).

### Statistical analysis

All the analyses were conducted using SAS 9.4 statistical software. Descriptive statistics included frequency and percent for categorical data, mean ± standard deviation for quantitative data. Statistical associations with impulsivity traits were analysed by nonparametric Mann–Whitney or Kruskal–Wallis test for categorical variables (including pairwise post-hoc comparisons of ranks), and Spearman rank correlation coefficient (r_S_) and test for quantitative variables. The analyses did not control for treatment status (simvastatin vs placebo) because, as previously said, abstinence or smoking reduction were not significantly different in both groups.

## Results

### Descriptive statistics

Among the ADDICSTATINE study participants, 68 were included in this study. Table 1 presents the demographics and smoking status characteristics of the participants. Overall, the mean age of participants was 45.6 years, ranging from 22.8 to 70.0 years, the majority being female (54.4%), married or cohabiting (61.8%), and currently working (86.8%).

**Table 1:** Sociodemographic and smoking characteristics of the total sample (n = 68).

Mean (standard deviation) [min; max] Or N (percent)	
Sociodemographic characteristics	
Age (year)	45.6 (10.2) [22.8 ; 70.0]
Sex	
Women	37 (54.4%)
Marital status	
Married/Cohabiting	42 (61.8%)
Education	
Junior high school	22 (32.4%)
Senior high school	19 (27.9%)
Undergraduate or higher	27 (39.7%)
Working (yes)	59 (86.8%)
Smoking characteristics at baseline	
Average number of cigarettes a day	20.3 (9.3) [10; 50]
Motivation score (Richmond test)	7.9 (1.5) [5; 10]
Tobacco craving (FTCQ-12 score)	3.92 (0.93) [1.92; 5.81]
Tobacco dependence (FTCD score)	
Mild (FTCD < 3)	11 (16.2%)
Moderate (FTCD > = 3 and < 8)	51 (75.0%)
Severe (FTCD > = 8)	6 (8.8%)
Smoking characteristics at 3 months	
Average number of cigarettes a day	6.1 (6.2) [0; 23]
Tobacco craving (FTCQ-12 score)	2.60 (1.08) [1.00; 4.88]
Smoking Status	
100% abstinence	18 (26.5%)
50–99% Reduction	32 (47.1%)
< 50% Reduction	18 (26.5%)
Smoking characteristics at 12 months	
Average number of cigarettes a day	12.5 (7.2) [0–32]
Smoking Status	
100% abstinence	18 (26.5%)
50–99% Reduction	19 (27.9%)
< 50% Reduction	31 (45.6%)
UPPS-P	
Negative urgency (n = 65)	9.46 (2.14) [4–15]
Positive Urgency	10.35 (2.18) [6–16]
Lack of premeditation	7.65 (2.12) [4–15]
Lack of perseverance (n = 67)	6.85 (2.19) [4–16]
Sensation seeking	9.63 (2.23) [5–16]
Evolution in smoking characteristics between baseline, 3 months and 12 months later	
Percentage of reduction in the number of cigarettes smoked per week between baseline and 3 months later	68.0 (30.0) [0 ; 100]
Percentage of reduction in the number of cigarettes smoked per week between baseline and 12 months later	49.3 (43.2) [− 50; 100]
Evolution of FTCQ-12 score between baseline and 3 months later	− 1.32 (1.29) [− 4.56; 1.15]

*FTCD* Fagerström Test for Cigarette Dependence, *FTCQ-12* French Tobacco Craving Questionnaire-12, *T0* baseline (just before the beginning of the cessation attempt), *T3* 3 months after the beginning of the cessation attempt, *T12* 12 months after the beginning of the cessation attempt.

At baseline, participants smoked on average 20.3 ± 9.3 cigarettes per day, had, for three quarters of them (75.0%), a mild dependence score according to the FTCD and low craving scores evidenced by the mean FTCQ-12 (3.9 ± 0.9). The percentage of reduction in weekly cigarette consumption was 68.0% from baseline to month three and 49.3% from baseline to month 12 after smoking cessation. At month 12, 18 (26.5%) participants were abstinent, 19 (27.9%) had reduced their consumption by 50% or more and 31 (45.6%) had a reduction in smoking < 50%. The 5 different dimensions of impulsivity did not differ significantly between the 2 groups (simvastatin and placebo) of the ADDICSTATINE study (supplementary data).

### Association between impulsivity traits at the end of the smoking cessation attempt and smoking cessation success

At 12 months, abstainers and participants who reduced smoking by 50% or more had significantly lower scores in negative and positive urgency compared to participants who reduced smoking by less than 50% (*p* = 0.011 and 0.0059 respectively, Table 2). Indeed, positive and negative urgency traits scores at 12 months were significantly and negatively correlated with smoking reduction at 12 months (*p* = 0.017 and 0.0012, Table 3): the lower the positive and negative urges, the higher the percentage reduction. The three other impulsivity traits (i.e., sensation seeking, lack of premeditation and lack of perseverance) were not associated with smoking cessation success at 12 months.

**Table 2:** Association between impulsivity traits and smoking cessation success.

	Negative urgency (n = 65)	Positive urgency (n = 68)	Lack of premeditation (n = 68)	Lack of perseverance (n = 67)	Sensation seeking (n = 68)
Mean (standard deviation) [min–max] (n)					
Smoking status at 3 months					
100% Abstinence (n = 18)	9.13 (2.13) [6–14](n = 16)	9.56 (2.12) [6–14]	7.61 (1.65) [4–12]	6.29 (1.72) [4–9] (n = 17)	9.06 (2.82) [5–16]
50–99% Reduction (n = 32)	9.00 (2.11) [4–15]	10.22 (2.15) [6–15]	7.34 (1.77) [4–11]	6.88 (2.04) [4–12]	9.75 (2.02) [6–15]
< 50% Reduction (n = 18)	10.65 (1.87) [8–15](n = 17)	11.39 (1.97) [8–16]	8.22 (2.96) [4–15]	7.33 (2.77) [4–16]	10.00 (1.91) [7–14]
*p*	0.017**	0.039***	0.46	0.43	0.43
Smoking status at 12 months					
100% Abstinence (n = 18)	8.88 (2.00) [6–14] (n = 16)	9.39 (2.06) [6–14]	7.44 (1.10) [5–9]	6.59 (1.58) [4–9] (n = 17)	9.17 (2.60) [5–16]
50–99% Reduction (n = 19)	8.68 (1.67) [6–11]	9.79 (2.04) [6–14]	7.84 (2.12) [9–14]	7.16 (2.39) [4–12]	9.32 (1.92) [5–12]
< 50% Reduction (n = 31)	10.27 (2.26) [4–15] (n = 30)	11.26 (2.02) [7–16]	7.65 (2.58) [4–15]	6.81 (2.39) [4–16]	10.10 (2.15) [6–15]
*p*	0.011**	0.0059**	0.81	0.92	0.28

**< 50% Reduction and 100% abstinence: Significant at 0.05 level— < 50% Reduction and > 50% Reduction: Significant at 0.05 level.

***< 50% Reduction and 100% abstinence: Significant at 0.05 level.

**Table 3:** Correlations between impulsivity traits and smoking status at 3 and 12 months.

	Negative urgency (n = 65)	Positive urgency (n = 68)	Lack of premeditation (n = 68)	Lack of perseverance (n = 67)	Sensation seeking (n = 68)
Spearman correlation coefficients					
Tobacco craving at 3 months (FTCQ-12 score)	r_s_ = 0.17 *p* = 0.16	**r**_**s**_** = 0.29** ***p* = 0.015**	r_s_ = 0.17 *p* = 0.17	r_s_ = 0.094 *p* = 0.45	r_s_ = 0.23 *p* = 0.055
Number of cigarettes smoked per day at 3 months	r_s_ = 0.23 *p* = 0.070	**r**_**s**_** = 0.27** ***p* = 0.024**	r_s_ = 0.11 *p* = 0.38	r_s_ = 0.10 *p* = 0.41	r_s_ = 0.13 *p* = 0.30
Number of cigarettes smoked per day at 12 months	r_s_ = 0.20 *p* = 0.16	**r**_**s**_** = 0.37** ***p* = 0.0068**	r_s_ = 0.19 *p* = 0.17	r_s_ = 0.10 *p* = 0.48	r_s_ = 0.027 *p* = 0.85
% of reduction in the number of cigarettes smoked per week between baseline and 3 months	**r**_**s**_** = **− **0.25** ***p* = 0.047**	r_s_ = − **0.28** *p* = **0.021**	r_s_ = − 0.062 *p* = 0.62	r_s_ = − 0.12 *p* = 0.33	r_s_ = − 0.13 *p* = 0.28
% of reduction in the number of cigarettes smoked per week between baseline and 12 months	**r**_**s**_** = **− **0.29** *p* = **0.017**	r_s_ = − **0.39** *p* = **0.0012**	r_s_ = − 0.092 *p* = 0.46	r_s_ = − 0.087 *p* = 0.48	r_s_ = − 0.014 *p* = 0.26

*FTCD* Fagerström Test for Cigarette Dependence, *FTCQ-12* French Tobacco Craving Questionnaire-12, *T0* baseline (just before the beginning of the cessation attempt), *T3* 3 months after the beginning of the cessation attempt, *T12* 12 months after the beginning of the cessation attempt.

Similar results were also found at three months. Indeed, patients who were abstinent three months after the initial quit attempt had also lower negative and positive urgency (*p* = 0.017 and 0.0039 respectively, Table 2). Negative urgency and positive urgency were significantly and negatively correlated with the percentage of reduction in the number of cigarettes smoked between baseline and three months (*p* = 0.047 and 0.021, Table 3). Sensation seeking, lack of premeditation and lack of perseverance scores were not associated with smoking cessation success at three months.

Positive urgency was positively correlated with higher craving at the end of treatment (*p* = 0.015, Table 3), while the four other impulsivity traits were not.

### Other factors associated with impulsivity traits at 12 months after the smoking cessation attempt

Positive urgency was negatively associated with marital status, with higher score among single individuals, but not with age, education status, nor professional status (Tables 2 and 3). Negative urgency was associated with none of the sociodemographic characteristics. Sensation seeking differed significantly between men and women, with men reporting higher levels of sensation seeking (Table 2). Sensation seeking also differed significantly by education level (senior high school and undergraduate or higher are significantly different). Lack of premeditation and lack of perseverance were significantly and negatively correlated with baseline motivation to quit (Table 3).

## Discussion

In this study, smokers with higher positive urgency and higher negative urgency had lower rates of smoking cessation success and a lower reduction in the number of cigarettes smoked 12 months after the beginning of the smoking cessation attempt. These impulsivity traits were also associated with the smoking cessation success at three months. In contrast, we found that smoking cessation success at three and 12 months was not associated with the other impulsivity traits, sensation seeking, lack of premeditation or lack of perseverance.

Evidence from cross-sectional studies suggests that negative and positive urgency were both associated with heavier use and tobacco dependence^28–30^. Higher impulsivity has been associated with difficulty quitting smoking in adults, smokers with higher levels of trait-impulsivity relapsing more quickly than those with lower levels following a one-day smoking cessation workshop without pharmacological intervention^8^. A meta-analysis of 97 studies examined the relationships between these specific impulsivity-related traits and smoking status (dependent / nondependent / chippers smokers versus never smokers / ex-smokers,^19^). They demonstrated that both adult smoking status and nicotine dependence were weakly but significantly associated with each of the five impulsive traits, positive urgency and lack of premeditation showing better at differentiating smokers from non-smokers.

Our main result is that the two main impulsivity traits associated with smoking cessation success were positive and negative urgency. Our results are congruent with previous research on the personality dimensions or traits associated with addictive disorders (in this case, lower conscientiousness / higher impulsivity)^31,32^, negative urgency being associated with worse substance use disorder outcome after psychotherapy^13^. These two impulsivity dimensions relate to how a given individual copes with positive or negative emotions, which is strongly linked to emotion dysregulation, a key risk factor for substance-related disorders and other psychiatric disorders^33^. Interestingly, our results are in line with the recent proposal by some authors that both positive and negative urgency are emotion-related impulsivity traits that cohere as a single cluster of items termed “general urgency”^34^. This general urgency could be defined as the “tendency to act rashly in intense emotional contexts” and it is thought to be a transdiagnostic risk factor in psychopathology^34^. Our results are also consistent with the idea that smokers may be a heterogenous population and that interindividual differences in personality may impact the trajectory of their cigarette consumption.

In this study, we also found no association between smoking cessation success and both sensation seeking, lack of premeditation or lack of perseverance. The lack of association between smoking cessation success and sensation seeking may be surprising, as sensation seeking is a known risk factor for substance use^35,36^. However, we may explain our results by two hypotheses. First, sensation seeking is more important for tobacco initiation than for quitting^37,38^. Indeed, the recruited patients were relatively older smokers, who have been smoking for a long time. For them, there would not be any element of novelty in smoking and therefore sensation seeking may be less relevant in predicting their smoking behavior, compared to younger individuals who could smoke because of the novelty of the smoking experience and the positive reinforcement they receive from smoking^39^. Second, nicotine use is a powerful mood regulator^40^ and smoking to alleviate negative mood states is a common motivation for smokers^41^. Given these pharmacological properties of tobacco and nicotine, smokers may present high prevalence for mood and anxiety disorders as demonstrated with other substance use disorders, and these disorders are associated with positive and negative urgency. However, they may exhibit lower sensation seeking scores than patients with other substance-related disorders such as cocaine use disorder or other illicit drug use disorder. In addition, sensation seeking is a risk factor for substance use, but, as opposed to other impulsivity traits, its contribution to the maintenance of the substance use despite negative consequence is not always found^13^. As proposed by Ersche in stimulant-dependent individuals, impulsivity may be a behavioral endophenotype mediating the risk for stimulant dependence that may be exacerbated by chronic drug exposure, whereas abnormal sensation-seeking may be more likely an effect of stimulant drug abuse^42^.

At a practical level, our study highlights the need to provide a systematic and comprehensive assessment of the interindividual differences in terms of impulsivity when managing patients seeking treatment for smoking. In addition to the assessment of the severity of tobacco dependence, to its possible harms, and to the patient’s motivation to change his/her smoking behavior, assessing how a given patient copes with positive and negative emotions may provide additional treatment targets based on his/her personality profile. In treatment seeking smokers, emotion dysregulation may be a transdiagnostic construct that may be helpful for patients both at a comprehensive level (i.e., provide them with a better understanding of why they may experience more difficulties when stopping tobacco, thus leading to lower learned helplessness and higher self-esteem) and at a practical level (i.e., provide them with adequate psychotherapeutic treatments targeting emotion dysregulation). Future studies should assess what could be the most effective interventions to target these impulsivity dimensions, and how such interventions may impact treatment outcome. If confirmed, this could lead to a better integration of psychological treatments targeting emotion regulation to conventional smoking cessation strategies, providing a more comprehensive biopsychosocial approach for these patients. This could be easily done in clinical practice by proposing an integrative Cognitive Behavioral Therapy that could include emotion-regulation skills (i.e., a process-based therapy) in addition to the application of classical behavioral principles (i.e., psychotherapy based on classical and operant conditioning principles) and cognitive principles (i.e., psychotherapy based on cognitive and social-cognitive principles)^43^. This is in line with Bradizza et al., who demonstrated that among pregnant smokers, an emotion-regulation treatment plus a cognitive behavioral therapy was more effective than a standard cognitive-behavioral smoking cessation therapy in providing smoking abstinence^44^. Finally, in a patient with high positive or negative urgency, a systematic assessment (and treatment if screened positive) of the psychiatric disorders usually associated with emotion dysregulation may be helpful (i.e., major depressive disorder, bipolar disorder, anxiety disorders, post-traumatic stress disorder, but also attention-deficit/hyperactivity disorder or personality disorders)^45,46^. Long-term tobacco consumption does indeed increase anxiety and depressive symptoms, but data on the evolution of depressive symptoms after smoking cessation are contradictory, with either stabilization or improvement^47^. However, we lack data on the evolution of symptoms of other psychiatric disorders following smoking cessation. A joint study of the evolution of psychiatric disorders and emotional dysregulation after smoking cessation would help to clarify the exact contribution of each of these disorders.

The current study has some limitations. First, impulsivity was assessed at 12 months only. However, impulsivity is assumed to be rather stable over time^13^. Future studies assessing impulsivity at the beginning of the study are needed to replicate our results. Moreover, smoking abstinence at T12 was only self-reported abstinence by participant and was not CO validated. Another limitation of the study is the selection bias. Indeed, recruited patients were smokers that voluntarily engaged in a smoking cessation attempt and were motivated to quit tobacco. Thus, our results may not generalize to patients that are not already in an advanced stages of the process of changing their tobacco habits^48^. In addition, we excluded smokers with co-occurrent addictive disorders. Thus, future work should verify that the same impulsivity traits predict relapse in patients with co-occurring addictive disorders and dual disorders. Another limitation is the scale used to measure impulsivity, which is a self-administered questionnaire, which may lead to social desirability biases. Complementary experimental objective measures of impulsivity would be helpful. Lastly, sample size that did not allow us to use multivariable analyses to adjust for potential confounding factors.

## Conclusion

The present study suggests that personality characteristics such as impulsivity traits, and more specifically positive and negative urgency, are associated with smoking cessation success and failure. These findings are important because elucidating psychological characteristics implicated in smoking treatment outcome may ultimately help in identifying the patients that are at higher risk of relapse and in the design of better, tailored treatment regimens to prevent relapse. Intervention strategies that target these impulsive traits and emotion dysregulation in combination with conventional treatment are also needed to show whether they improve smoking cessation success.

### Supplementary Information

Supplementary Information.

## Data Availability

The datasets generated during and/or analysed during the current study are available from the corresponding author on reasonable request.
