# Supplementary material for: Smokers with higher positive or negative urgency have lower rates of smoking cessation success 12 months after a quit attempt
Source: Sci Rep. 2024 May 29;14:12321. doi: 10.1038/s41598-024-62972-6 (PMC11137130; doi:10.1038/s41598-024-62972-6)
Supplement: Supplementary file 1 — Supplementary Information. [file 41598_2024_62972_MOESM1_ESM.docx]

Supplementary data - Impulsivity traits of the total sample at 12 months according to treatment group

|  | Simvastatine | Placebo | p |
| --- | --- | --- | --- |
| Negative urgency | 9.17 (2.24) [4-15] n=35 | 9.80 (2.01) [6-15] n=30 | 0.32 |
| Positive Urgency | 10.23 (2.03) [6-16] n=35 | 10.48 (2.35) [6-15] n=33 | 0.46 |
| Lack of premeditation | 7.83 (2.28) [4-15] n=35 | 7.45 (1.95) [4-12] n=33 | 0.72 |
| Lack of perseverance | 7.03 (2.33) [4-16] n=35 | 6.66 (2.04) [4-12] n=32 | 0.60 |
| Sensation seeking | 9.86 (2.30) [5-16] n=35 | 9.39 (2.15) [5-13] n=33 | 0.66 |
